# Head and Neck Kaposi Sarcoma—An Updated Focus on Clinical and Epidemiological Characteristics: A Comprehensive Review

**DOI:** 10.3390/diseases14030084

**Published:** 2026-02-24

**Authors:** Luis Alberto Gaitán-Cepeda, Brenda Daniela Ortega-Hidalgo, César Esquivel-Chirinos, Iñigo Gaitán-Salvatella, Stephany Paladines-Calle, Daniela Carmona-Ruíz

**Affiliations:** 1 Department of Oral and Maxillofacial Medicine and Pathology, Research and Graduate Division, Dental School, National Autonomous University of Mexico, Mexico City 04510, Mexico; bdaniela.ortegah@gmail.com; 2 Basic Sciences Area, Professional Studies Division, Dental School, National Autonomous University of Mexico, Mexico City 04510, Mexico; cesaresquivelch@fo.odonto.unam.mx; 3 Pharmacy Department, CEU Cardenal Herrera University, Alfara del Patriarca, 46115 Valencia, Spain; inigo.gaitansalvatella@uchceu.es; 4 Dental School, Polytechnic Salesiana University, Cuenca 010101, Ecuador; spaladines@ups.edu.ec; 5 Orthodontics Area, Professional Studies Division, Dental School, National Autonomous University of Mexico, Mexico City 04510, Mexico; danielacr@fo.odonto.unam.mx

**Keywords:** Kaposi sarcoma, oral Kaposi’s sarcoma, HHV-8, KS/HHV8, oral cavity, epidemiological forms

## Abstract

Background/Objectives: Interest in Oral Kaposi’s sarcoma (OKS) has declined recently, potentially causing diagnostic errors due to physicians’ unfamiliarity with its presentation. This review describes clinical and demographic characteristics of OKS patients across epidemiological groups. Methods: A literature search of studies published from 1957 to December 2024 was conducted using PubMed, Web of Science, Cochrane Library, Scopus, and Google Scholar. Studies with confirmed oral Kaposi sarcoma were included, while those with incomplete data were excluded. Cases were grouped into classic, endemic, epidemic (AIDS-related), iatrogenic, and HIV-negative males who have sex with males. Sex distribution, mean age, clinical appearance, lesion topography, and cause-related information for iatrogenic forms were recorded. Results: A total of 1812 articles were identified through database search. During initial screening, 1162 articles were excluded as duplicates. Of the remaining 650 papers, 338 were dismissed based on title and abstract. Of the remaining 312 articles for full-text review, 93 could not be accessed, leaving 219 articles for analysis. After screening, 123 were excluded, resulting in 117 articles for review. These were categorized as: 16 classical KS, 7 endemic-African, 20 iatrogenic, 70 epidemic-HIV/AIDS-related, and four articles reporting cases among MSM not related to HIV infection. A total of 152 patients with OKS were analyzed. Mean age was 38.04 years (range, 2–86 years), and 75% were male. Of all cases, 64.4% were epidemic, 13.8% iatrogenic, 10.5% classical, and 4.6% endemic. The palate was most common (44.6% of lesions), followed by gingiva (25.3%). Nodular or papular presentations were most frequent. Conclusions. OKS occurs in all KS epidemiological forms, and since this tumor can mimic gingival and periodontal lesions, dentists and physicians must be alert to identify oral Kaposi’s sarcoma.

## 1. Introduction

Kaposi sarcoma (KS) is a multicentric vascular neoplasm of endothelial origin with low-grade malignancy. It was first described in 1872 by Hungarian dermatologist Moritz Kaposi [1]. KS presents as multiple purple-blue or reddish-brown plaques and nodules. It is a rare neoplasm associated with Mediterranean and young African populations, particularly in sub-Saharan Africa [2]. In the 1980s, during the HIV pandemic, KS cases increased exponentially in HIV-positive individuals, becoming an AIDS-defining condition and the most significant HIV/AIDS-related malignancy [3]. AIDS-related KS cases led to a specific epidemiological variant: the AIDS-related or epidemic form. Because this form often involves the oral cavity, it is referred to as oral Kaposi’s sarcoma (OSK). Patients receiving immunosuppressive therapy, mainly for transplantation, develop OSK more frequently than immunocompetent patients. Four epidemiological variants of KS have been recognized: classic, endemic, epidemic, and iatrogenic/transplant-related [4]. Recently, a fifth variant was described in HIV-negative men who have sex with men (MSM) [5]. The classic form typically occurs in older adults, aged 50–70 years, primarily in Mediterranean and Eastern European males, and usually affects the skin of the lower extremities. One-third of patients with classic KS develop a second primary cancer, most commonly non-Hodgkin’s lymphoma (NHL). OSK is uncommon and presents as well-defined, painless, brownish-red to violaceous macules or papules on the hard palate or gums [6]. The endemic form includes African children and young HIV-seronegative males, mainly from sub-Saharan Africa. The epidemic form is the most common presentation of KS and is typically the most aggressive. KS was one of the first AIDS-defining diseases. It shows a strong preference for the oral cavity, which led to the term oral Kaposi’s [6]. Iatrogenic cases occur in immunocompromised individuals due to medical treatment. The incidence of KS in immunosuppressed renal transplant recipients is 150–200 times higher than that in the general population [7,8,9]. The fifth variant involves HIV-negative MSM. To date, there is scarce information about it, including a possible association with OSK [5].

Although the etiology of KS is multifactorial, Human Herpesvirus 8 (HHV-8) has been detected in more than 90% of KS lesions, irrespective of epidemiological differences. Although HHV-8 is the leading cause of KS, the exact mechanism remains unclear; cofactors such as host immunodeficiency are essential for tumor development [9]. HHV-8 was first identified as a novel herpesvirus in 1994 [10,11]. KS is an angiosarcoma characterized by the rapid growth of spindle cells of endothelial origin, incomplete vascular slit formation, and extravascular hemorrhage [9]. No histological differences have been identified between the KS epidemiological subtypes [9]. OKS has been reported in all epidemiological forms. Early OKS lesions are typically macular or nodular, single or multiple, and purple or reddish, and occur mainly on the palate and gingiva [6].

The clinical and demographic characteristics of OSK have been widely described, with a focus on epidemic patterns given its high prevalence. Current generations may not be accustomed to identifying and seeking timely diagnosis, particularly those born after the mid-1990s, when highly active antiretroviral therapy was introduced and gradually reduced the prevalence of OSK. However, the possible emergence of a new clinical variant unrelated to HIV infection or to iatrogenic procedures, such as in transplant recipients, but occurring in apparently healthy subjects, could lead to a diagnostic error and delay treatment. It is necessary to update this. However, it remains unclear whether the clinical features are shared across different epidemiological varieties of OSK. Therefore, this systematic review aimed to describe the clinical and demographic characteristics of patients with OSK reported in the literature and identify commonalities across epidemiological groups.

## 2. Materials and Methods

A comprehensive scientific literature search was conducted from 1957 to December 2024, with no limits or filters, to answer the research question, “What are the clinical and epidemiological characteristics of patients with oral Kaposi sarcoma, particularly regarding the epidemiological varieties of Kaposi’s Sarcoma?” The databases searched included PubMed, Web of Science, Cochrane Library, Scopus, and Google Scholar. The search strategy was as follows: ((((oral) OR (oral cavity)) OR ((mouth) OR (oral mucosa))) OR (((tongue) OR (palate)) OR ((tonsil) OR (floor of the mouth)))) AND (((Kaposi sarcoma) OR (HHV8)) OR (KSHV)). For the endemic form, the search strategy was: (((Africa) OR (sub-Saharan Africa)) AND (((child) OR (children)) AND (((Kaposi sarcoma) OR (HHV8)) OR (KSHV)))) AND ((((oral) OR (oral cavity)) OR ((mouth) OR (oral mucosa))) OR (((tongue) OR (palate)) OR ((tonsil) OR (floor of the mouth)))).

Inclusion Criteria: All papers published in English or Spanish reporting cases with a histopathologically confirmed diagnosis of oral Kaposi sarcoma, regardless of sex and age, from January 1957 to December 2024, were considered. To qualify, the case report must describe a primary KS located in the oral cavity, including the buccal mucosa, tongue, gums, palate (hard and soft), floor of the mouth, intraosseous lesions (maxillary or mandibular), or tonsils. Exclusion Criteria: Theses, conference proceedings, books, or atlas were excluded. Cases with only clinical diagnoses (without histopathological confirmation) were excluded. Studies with unclear abstracts, full texts, or incomplete clinical or demographic data were excluded from the analyses. Cases of oral metastasis from primary systemic Kaposi’s sarcoma were excluded. Outcome Measures: All selected cases were grouped by demographic and clinical characteristics as follows: classic or Mediterranean form; epidemic or AIDS-related; iatrogenic or post-transplant; endemic or African; and HIV-negative MSM form [5,12]. In each group, the sex distribution; mean age; clinical appearance of lesions; lesion topography; and, in the iatrogenic group, the cause-related factors were recorded.

Two researchers (LAGC and BDOH) independently assessed the quality of the studies and compared their results. If they disagreed, a third researcher (SPC) was involved in the discussion to reach a consensus.

IBM SPSS Statistics version 22.0 was used for the statistical analyses. The chi-square test was used to assess the relationship between sex and epidemiological forms. The mean age among patients with KS in the epidemiological forms was compared using a *t*-test. In both analyses, the significance level was set at *p* = 0.05 (_IC95%_).

## 3. Results

**Figure 1:**
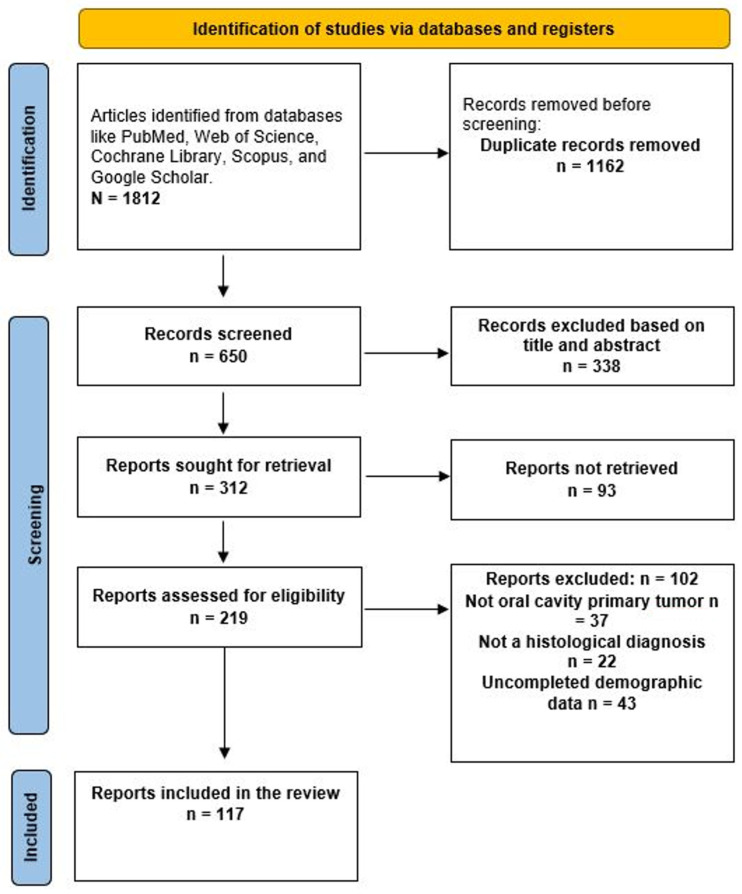
Prisma diagram to identify eligible papers.

A total of 1812 articles were identified through an advanced database search. During the initial screening to remove duplicates, 1162 articles were excluded. Of the remaining 650 papers, 338 were excluded based on their title and abstract. Of the remaining 312 articles, 93 could not be accessed, leaving 219 articles eligible for analysis. A total of 219 articles were screened; 123 were excluded for various reasons, leaving 96 articles for inclusion in the review (Figure 1). The 117 articles were categorized as follows: 16 as classical KS [4,6,13,14,15,16,17,18,19,20,21,22,23,24,25,26], 7 as endemic-African [27,28,29,30,31,32,33], 20 as Iatrogenic or transplant-related [24,34,35,36,37,38,39,40,41,42,43,44,45,46,47,48,49,50,51,52], 70 as epidemic-HIV/AIDS-related [2,5,7,53,54,55,56,57,58,59,60,61,62,63,64,65,66,67,68,69,70,71,72,73,74,75,76,77,78,79,80,81,82,83,84,85,86,87,88,89,90,91,92,93,94,95,96,97,98,99,100,101,102,103,104,105,106,107,108,109,110,111,112,113,114,115,116], and four articles reported cases among MSM not related to HIV [8,117,118,119].

**Figure 2:**
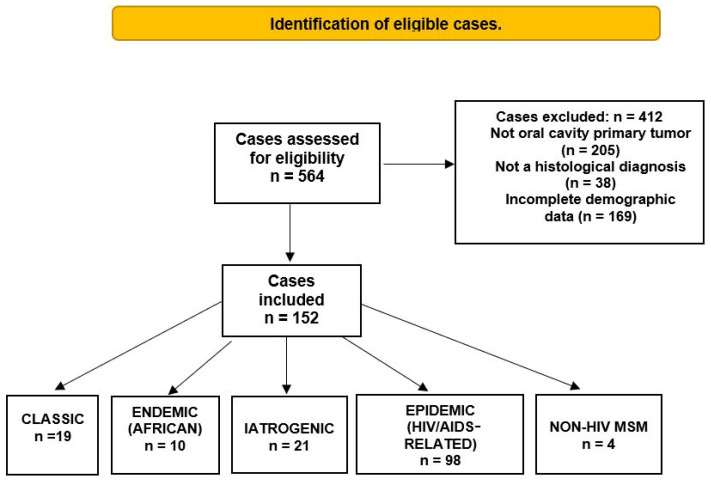
Identification and distribution of oral Kaposi’s sarcoma cases regarding the epidemiological form.

Of the 117 papers included, 152 cases of OKS met the inclusion criteria and were analyzed in this review (Figure 2). The mean age of the patients was 39.9 years (SD = 18.4; range, 2–86 years). Most patients with OKS were male (111 cases, 73%), whereas females were less frequently affected (29 cases, 19%). The sex of the patients was not specified in 12 cases (7.8%). The most frequently reported variety was epidemic, accounting for 98 cases (64.4%) [2,5,7,53,54,55,56,57,58,59,60,61,62,63,64,65,66,67,68,69,70,71,72,73,74,75,76,77,78,79,80,81,82,83,84,85,86,87,88,89,90,91,92,93,94,95,96,97,98,99,100,101,102,103,104,105,106,107,108,109,110,111,112,113,114,115,116]. The iatrogenic variant was found in 21 cases (13.8%) [24,34,35,36,37,38,39,40,41,42,43,44,45,46,47,48,49,50,51,52], followed by the classical variety in 19 cases (12.5%) [6,13,14,15,16,17,18,19,20,21,22,23,24,25,26]. The endemic African variety had ten cases (6.5%) [27,28,29,30,31,32,33], and the least reported was MSM non-HIV, with four cases (2.6%) [117,118,119].

The epidemiological form of KS with the highest mean age was the classic form, at 69.8 years (SD ± 17.4), whereas the HIV-related, iatrogenic, and non-HIV-MSM varieties had virtually the same mean ages: 37, 40.3, and 38.5 years, respectively. As expected, the lowest mean age was observed for the endemic/African form of the disease. However, the classic form was the only one in which women predominated (57.8%). In contrast, in the HIV-related variety, the male-to-female ratio was approximately 7:1. The endemic variety was characterized by a considerable lack of clinical-demographic data, making any inference speculative. We identified only four cases of OSK in the non-HIV-MSM form; therefore, no analysis could be performed. All data are presented in Table 1.

### 3.1. Classic Form

Nineteen cases were classified as classical OKS [6,13,14,15,16,17,18,19,20,21,22,23,24]. Eleven patients were female and eight were male, with an average age of 69.8 years (SD ±14.7; range, 24–86 years). The most frequent locations of OKS lesions were the palate and gums. The most common clinical feature was tumor-related, with seven cases reported. No statistically significant association was found between the clinical and demographic variables [4,6,9,13,14,15,16,17,18,19,20,21,22,23,24,25,26].

### 3.2. Endemic Form

Ten cases of OKS associated with endemic-OKS have been reported in the scientific literature [25,26,27,28,29,30], with an average age of 4.6 years (±1.7), ranging from 2–6 years. These cases provided the least amount of clinical and demographic data. Only one case indicated gender, which was male. The location was described in six cases, with three on the palate, one on the gingiva, one on the buccal mucosa, and one intraosseous. Three cases were defined as tumor growth, while the remaining seven reported only an increase in volume or swelling [27,28,29,30,31,32,33].

### 3.3. Iatrogenic Type

There were 21 cases, with a mean age of 40.3 years (SD ±14.9; range, 21–68 years). These cases were associated with transplantation or non-transplant-related immunosuppressive treatment [24,34,35,36,37,38,39,40,41,42,43,44,45,46,47,48,49,50,51,52]. Among the transplanted patients, there were 13 cases, primarily involving kidney transplantation. Regarding immunosuppression not related to transplantation, three cases were associated with pemphigus vulgaris treatment, two with lymphoma treatment, one with OKS related to leprosy treatment, and one with calcium channel blocker use. More males were reported, accounting for 14 of the 21 cases included in the study. Topographically, the gums and palate had the highest number of cases, with six and five cases, respectively. The most frequently observed clinical lesions were nodular–papular, accounting for nine cases.

### 3.4. Epidemic HIV-Related Form

The KS form with the highest number of OKS cases, totaling 98, accounts for 64.4% of all reported OKS cases [2,5,7,53,54,55,56,57,58,59,60,61,62,63,64,65,66,67,68,69,70,71,72,73,74,75,76,77,78,79,80,81,82,83,84,85,86,87,88,89,90,91,92,93,94,95,96,97,98,99,100,101,102,103,104,105,106,107,108,109,110,111,112,113,114,115,116]. The mean age was 37.04 years (SD ±11.7), with a range of 18–81 years old. Of these 98 patients, 87 (82.6%) were men. In terms of location, the palate and gingiva were the most common sites, with 50 and 16 cases, respectively. The most frequent clinical appearance was nodular–papular, observed in 39 of 98 cases. In the epidemiological analysis, the association with males was statistically significant (*p* < 0.05; odds ratio = 10, 95% CI = 4.3–23.3). The male:female ratio was 1:7. Similarly, the ratio of palate and OKS in this type was statistically significant (*p* < 0.05; odds ratio 2.3, 95% CI, 1.1–4.6). In the epidemic variety, the group was subdivided by age to examine possible differences or associations between clinical and demographic characteristics. Thus, cases < 40 years of age were grouped together, and cases ≥ 40 years of age were grouped separately. The <40-year group comprised 51 cases aged 18–39 years (mean age 31 years; SD ±5), while the ≥40-year group comprised 33 cases aged 41–81 years (mean age 50 years; SD ±10). No differences were found between the two age groups in the topographic distribution or clinical presentation of OKS lesions.

### 3.5. MSM-No Related HIV Type

Four reported cases related to this newly proposed variety were identified in the review [8,117,118,119]. The mean age of the patients was 38.5 years (±10.5), with a range of 28 to 53 years. Three lesions were found on the gingiva, and two were found on the palate. Two lesions were described as nodular–papular, and two were tumor-like. The small sample size precluded statistical analysis.

In all epidemiological forms, there were more lesions than cases because a single case could have multiple lesions. Table 2 presents all clinical data collected for the clinical variables included in each type of OKS.

## 4. Discussion

This systematic review examined the epidemiological and clinical characteristics of individuals with oral Kaposi’s sarcoma (OKS). OKS gained prominence four decades ago because of its high prevalence among individuals with AIDS, leading to extensive documentation during the epidemic. However, the introduction of highly active antiretroviral therapy (ART) has significantly reduced its prevalence, eventually leading to its disappearance in patients with access to ART. Consequently, dentists and physicians may not be familiar with KS clinical presentations, which can result in diagnostic errors when identifying classic OKS lesions. Five clinical and epidemiological KS subtypes have been proposed: classic, endemic, HIV-associated, iatrogenic/transplant-associated, and KS related to men who have sex with men without HIV+ [7,8]. It remains unclear whether the clinical characteristics of OKS are consistent across subtypes or vary by subtype. This systematic review demonstrates that OKS is present in all epidemiological forms, with varying prevalence rates. OKS is rare in HIV-negative patients, accounting for 18% of cases, whereas HIV-related OKS comprises 64.4% of published reports. Gender distribution varies; in HIV, iatrogenic, and non-HIV MSM forms, males are more frequently affected, with ratios ranging from 1:7 (F:M) in HIV-related cases to 1:1.7 (F:M) in the iatrogenic form. Only the classic form shows a higher proportion of females than males.

The limited number of reported cases of OKS in endemic and non-HIV MSM suggests cautious interpretation until more cases are analyzed. The results indicated that 73% of the reported cases involved men. However, these data are not representative, as only two of the 10 endemic patients included in this review reported their sex and age. It is difficult to identify a single justification for this lack of data. Primary oral Kaposi’s may be uncommon, but KS is not rare in Africa. Oral mucosal involvement is uncommon in children. Moreover, OKS is rare in children with HIV/AIDS. On the other hand, the survival rate may be very low among pediatric patients with KS; therefore, the presence of oral lesions may be of secondary importance or reflect underreporting rather than non-occurrence. In contrast, only four cases of non-HIV MSM were identified and included. The low number of OKS cases in this epidemiological group raises questions. The fact that it occurs exclusively in males is clearly related to the route of transmission and the subjects’ habits, rather than to a virus–host biological cause. The same applies to the epidemic form. To date, there have been few reported cases of OSK in non-HIV MSM subjects, and more reports are needed to conduct a more in-depth analysis.

In East Africa, a decline in the male-to-female ratio of KS has been reported, with more OKS cases among Tanzanian women, resulting in a male-to-female ratio of 1:2.3 [102]. This gender shift may be related to the sexual transmission of human herpesvirus 8 (HHV-8) in the sub-Saharan region via genital–oral or oral–oral routes, potentially influencing KS development [102]. Further reports on OKS in sub-Saharan populations are needed to verify this hypothesis.

Our findings identified two prevalent sites: the palate and gums, with epidemiological patterns suggesting increased susceptibility of the palate [102]. Most cases of oral Kaposi’s sarcoma (OKS) are characterized by nodular–papular localized lesions, indicating that OKS may represent a distinct clinical entity, as previously proposed [102]. Classic Kaposi’s sarcoma is a rare vascular tumor that manifests as slow-growing lesions on the extremities of older men. In long-standing cases, visceral organs may be involved [16]. Our results indicate that classic OKS predominantly occurs on the palate or tongue in women in their seventies and presents with a nodular or papular appearance. Classic OKS can invade the bone, resulting in tooth mobility. Morbidity may include pain, bleeding, and functional impairment [18]. OKS is rare in endemic KS. In our findings, endemic OKS accounted for 6.5% of cases, consistent with the previously reported 5% [25,26,27,28,29,30]. Although an extensive cohort study of African HIV-positive children with KS reported an OKS prevalence of 21–58% among patients [103], all cases involved children aged <6 years. Only one article specified the patient’s sex as male. The primary site for OKS was the palate, with three out of four cases occurring there. From an epidemiological perspective, endemic cases are intriguing. The demographic profile included children and young adults from sub-Saharan countries. However, the high prevalence of HIV/AIDS among children in this region may contribute to both epidemiological forms: endemic and epidemic, with overlap.

The primary causative agent of Kaposi’s sarcoma (KS) is human herpesvirus 8 (HHV-8), and immunodeficiency is often a necessary condition for its development [92]. Studies have shown that African children are more vulnerable to HHV-8 infection [102,103], particularly in Eastern and Central Africa, where the childhood HHV-8 infection rates are the highest. The widespread presence of both HHV-8 and HIV in these regions likely contributes to the high incidence of endemic KS, making it a common pediatric cancer in Africa [30]. El-Mallawany et al. [30] reported a high prevalence of oral Kaposi’s sarcoma (OKS). However, stage 1 of the Lilongwe Pediatric KS Staging Classification, which includes mild-to-moderate disease limited to the skin and oral mucosa, is relatively rare among African children with KS, comprising only 5–8% of cases [104], which is consistent with our findings. This apparent contradiction may stem from the criteria of our systematic review, which excluded case reports or series lacking comprehensive demographic data, thus omitting larger studies from our analysis.

The epidemic form of KS is the most thoroughly researched, resulting in the highest number of reported OKS cases. In our systematic review, it accounted for 64.4% of all cases, with a notable male predominance (female-to-male ratio of 1:7.1). The palate and gums were the primary anatomical sites of involvement. The high incidence of AIDS-associated OKS may be due to a higher viral load in saliva than in other bodily secretions, possibly because oropharyngeal epithelial cells serve as a reservoir for HHV-8 [105]. The introduction of highly active antiretroviral therapy in the mid-1990s [106] led to a decline in the frequency and prevalence of OKS, nearly eradicating it in developed countries with universal ART coverage. Consequently, newer generations of clinicians may lack experience in diagnosing OKS, potentially leading to misdiagnosis. Iatrogenic form was the second most prevalent type of OKS, representing 13.1% of the cases. It accounts for 1% of all reported cases, predominantly affecting men in their fifth decade of life and involving the palate, gums, and tongue. The oral manifestation of Kaposi’ can resemble gingival hyperplasia, which is significant for patients on cyclosporine, a drug commonly prescribed to transplant recipients, as it is associated with generalized, erythematous, fibrotic gingival hyperplasia that could be mistaken for KS. Clinicians must be aware of these differential diagnoses. The development of KS requires both HHV-8 infection and immunosuppression, suggesting that HHV-8 transmission routes may include: (1) new infection during the post-transplant period due to the patient’s immunosuppressed state, or (2) reactivation of latent HHV-8 in donor organs. Therefore, the primary treatment for KS in transplant recipients involves reducing immunosuppression, followed by chemotherapy if necessary [37]. For HIV-seronegative men who have sex with men, cases may be classified as sporadic or classic, although an associated immunodeficiency has not been confirmed. In these instances, the transmission routes should be carefully considered.

The primary etiological agent of OKS is HHV-8 [107]. HHV-8 was isolated from a KS lesion and was designated as KS-associated herpesvirus (KSHV) [10]. The oncogenic process induced by HHV-8 involves manipulation of cell cycle regulation, apoptosis, and immune evasion. Human herpesvirus type 8 is a double-stranded DNA virus of 165–170 kilobases in the Gammaherpesvirinae family [120,121,122]. HHV-8 has five distinct genotypes (A–E) [120,121]. HHV-8 virions have an icosahedral capsid that encloses viral DNA and bears viral glycoproteins [123]. The life cycle of HHV-8 includes latency and lytic replication phases [124,125]. During latency, the viral genome persists as an episome within the nucleus, expressing limited viral genes, notably LANA-1, which maintains genome stability and evades immune detection [126,127,128]. The lytic phase, induced by immunosuppression or chemical agents, involves viral gene expression and the production of virions. vGPCR activates pro-inflammatory pathways, contributing to angiogenesis and tumorigenesis [126], whereas ViL6 promotes cell proliferation and immune evasion [127]. VBCl-2 and ViaP are viral homologs of human anti-apoptotic proteins that promote the survival of infected cells [128]. LANA-1 interacts with retinoblastoma protein (pRb) and transcription factors (E2F). This phase reprograms the microenvironment, promoting cell proliferation and oncogenesis [120,128]. HHV-8 lytic activation, with increased viral load and elevated IL-6 and IL-10 levels, drives oncogenesis [129]. These actions promote cell proliferation and inhibit cell death in HHV-8-associated neoplasia [126,130].

Although the transmission pathways of HHV-8 are not fully elucidated, they appear to be intricately linked to the epidemiological classification of Kaposi’s sarcoma (KS) and its variants. Transmission modes include horizontal, vertical (mother-to-child), and sexual transmission. In Western countries, HHV-8 is predominantly transmitted through sexual contact, particularly among MSM. Conversely, in developing regions, especially sub-Saharan Africa, alternative transmission routes are evident, notably those involving children, such as mother-to-child transmission. Molecular evidence suggests that standard household practices, such as sharing food or sauce plates, facilitate the transmission of saliva contaminated with HHV-8 [118]. Consequently, countries and regions with a high prevalence of HHV-8 are associated with a high incidence of KS. Sexual transmission is a prevalent route in areas with low-to-intermediate HHV-8 prevalence [119]. In both contexts, studies have demonstrated that viral shedding in the saliva is common [131]. HHV-8 is not ubiquitous in healthy populations, and its prevalence varies globally, with rates ranging from 46% in Nigeria to 38%. 7% in Uganda, and 37.5% in Zambia to 1.3–4.4% in the USA, Southeast Asia, and the Caribbean [131,132]. These data suggest that HHV-8 infection is more prevalent in areas where Classic and Endemic KS occurs and in populations with a high HIV prevalence. Consequently, multiple epidemiological types may coexist within the same population, such as Mediterranean Africans and sub-Saharan individuals, potentially leading to the formation of epidemiological subgroups in studies. The overlap of KS types is inevitable; for instance, the endemic form is prevalent among children and young adults in sub-Saharan Africa, particularly among African males. However, the high prevalence of pediatric AIDS in this region, driven by the HIV epidemic, has resulted in the overlap of HIV-related and endemic HIV-negative KS, making KS one of the most common childhood cancers in Eastern, Central, and Southern Africa [104]. In contrast, the prevalence of KS in HIV-infected children in the United States and Europe is extremely low [131]. HHV-8 infection is, in part, independent of HIV [132]. Our findings revealed ten cases of Endemic KS, accounting for 6.5% of all reported cases. Variants within epidemiological categories include cases of HIV-positive individuals with undetectable viral loads and high CD 4 counts who develop KS. The classification of these patients as epidemic, classic, no related-HIV MSM, or as a sub-classification related to inflammatory response syndrome remains complex due to the overlap in several cases.

Several limitations are associated with this systematic review. The case series discussed in this review does not encompass all reported instances of OKS in the literature. During the initial phase of the HIV epidemic, numerous OKS cases were documented, predominantly in extensive series that outlined the general characteristics of the group or were organized by demographic factors such as gender or age. Consequently, individual data for each case were not provided. Therefore, we cannot assert that our findings represent all cases.

A primary concern is the lack of uniformity in case descriptions, resulting in incomplete demographic data in numerous instances. The most pronounced heterogeneity was observed in clinical descriptions owing to the wide variety of morphological descriptions of clinical lesions, including papules, nodules, or simply swelling, necessitating cautious interpretation of findings regarding the most prevalent clinical form. Nonetheless, reports consistently indicate that initial oral lesions typically present as single or multiple nodular or papular lesions that may progress to tumorous growths if left untreated. Additionally, noteworthy cases or case series published in local journals may not be included in the mainstream databases.

Most cases are reported by researchers or clinicians. These clinical cases are often published in local journals, which, although they have strong scientific validity, have limited dissemination and are not indexed in large databases. To mitigate this potential bias, we included databases with broader coverage, such as Google Scholar. Consequently, particularly for the African/endemic type, the limited number of reported cases may be attributable to this factor and may not reflect the low incidence of oral cavity Kaposi’s sarcoma in the endemic variety.

## 5. Conclusions

Despite advances in medical science, interest in Oral Kaposi’s sarcoma (OKS) has declined, potentially reducing awareness of its identification among younger generations [133]. The clinical presentation and common topographical occurrence of OKS facilitate its early diagnosis. Consequently, a dentist or oral medicine specialist can diagnose oral Kaposi’s through clinical examination. Given the range of differential diagnoses, physicians must obtain histopathological confirmation of the diagnosis. Health professionals should remain cognizant of the risk groups affected by OKS, be vigilant for oral lesions, and recognize the potential overlap among epidemiological forms of OKS.

## Figures and Tables

**Table 1:** Demographical characteristics of included OSK cases.

Ks Form		Total	Classic	Endemic	Iatrogenic	HIV/Aids-Related	Non-HIV/Aids MSM
Papers		117	16	7	20	70	4
			[4,6,13,14,15,16,17,18,19,20,21,22,23,24,25,26]	[27,28,29,30,31,32,33]	[24,34,35,36,37,38,39,40,41,42,43,44,45,46,47,48,49,50,51,52]	[2,5,7,53,54,55,56,57,58,59,60,61,62,63,64,65,66,67,68,69,70,71,72,73,74,75,76,77,78,79,80,81,82,83,84,85,86,87,88,89,90,91,92,93,94,95,96,97,98,99,100,101,102,103,104,105,106,107,108,109,110,111,112,113,114,115,116]	[8,117,118,119]
Cases		152	19	10	21	98	4
		(100%)	(12.9%)	(6.5%)	(13.1%)	(64.4%)	(2.6%)
Gender	Female	29	11	----	7	11	----
		(24.7%)	(57.8%)		(33.3%)	(11.2%)	
	Male	115	8	2	14	87 *	4
		(73.0%)	(42.1%)		(66.6%)	(82.6%)	(100%)
	N/S	8		8			
AGE		38.04	69.8	4.6	40.3	37.04	38.5
		(±10.7)	(±14.7)	(±1.7)	(±14.9)	(±11.72)	(±10.5)
AGE By Gender	Female	48.5	68.1	----	41	33.4	----
		(±22.5)	(±17.4)		(±12.2)	(±17.6)	
	Male	39.9	71.8	2	39.9	37.5	38.5
		(±15.1)	(±11.3)		(±16.7)	(±10)	(±10.5)

[ ]—references; ±—standard deviation; (%)—prevalence; N/S—non-specified; *—*p* < 0.05, OR = 10 (IC 4.3–23.3).

**Table 2:** Clinical characteristics of included OSK cases regarding epidemiological type.

Type		Total (*n* = 152)	Classic (*n* = 19)	Endemic (*n* = 10)	Iatrogenic (*n* = 21)	HIV/Aids-Related (*n* = 98)	Non-HIV/Aids MSM (*n* = 4)
Topography	Palate	68	9	3	5	50 *	1
		(44.7%)	(47.3%)	(30%)	(23.8%)	(51%)	(20%)
	Gums	28	2	1	6	16	3
		(18.4%)	(10.5%)	(10%)	(28.5%)	(16.3%)	(60%)
	Tongue	15	4	----	3	8	----
		(9.8%)	(2.6%)		(14.2%)	(8.1%)	
	Tonsils	7	1	----	2	4	----
		(4.6%)	(5.2%)		(9.5%)	(4%)	
	Parotid	7	3	----	----	4	----
		(4.6%)	(1.9%)			(4%)	
	Buccal Mucosa	14	1	1	3	8	1
		(9.2%)	(5.2%)	(10%)	(14.2%)	(8.1%)	(20%)
	Intraosseous	13	----	1	----	12	----
		(8.5%)		(10%)		(12.2%)	
	N/S	10	----	4	3	1	----
		(6.5%)		(50%)	(14.2%)	(1%)	
Clinical Appearance	Macular	26	5	----	3	18	----
		(17.1%)	(26.3%)		(14.2%)	(18.3%)	
	Nodular/ Papular	55	5	----	9	39	2
		(36.1%)	(26.3%)		(42.8%)	(39.7%)	(50%)
	Tumoral	37	7	3	2	23	2
		(24.3%)	(36.8%)	(30%)	(9.5%)	(23.4%)	(50%)
	N/S	38	3	7	7	21	----
		(25%)	(15.7%)	(70%)	(33.3%)	(21.4%)	

(%)—Prevalence; N/S—Non specified; * chi square test—*p* < 0.05, OR = 2.3 (IC 1.1–4.6).

## Data Availability

No new data was created since only documentary research techniques and methods were used.

## Abbreviations

The following abbreviations are used in this manuscript:

KS	Kaposi’s Sarcoma
HIV	Human immunodeficiency virus
AIDS	Acquired Immunodeficiency Syndrome
OSK	Oral Kaposi’s sarcoma
MSM	Men who have sex with men
HHV-8	Human Herpesvirus 8
ART	Antiretroviral treatment
KSHV	Kaposi sarcoma Herpes Virus
